# The Intriguing Relationships of von Willebrand Factor, ADAMTS13 and Cardiac Disease

**DOI:** 10.3390/jcdd8090115

**Published:** 2021-09-15

**Authors:** Benjamin Reardon, Leonardo Pasalic, Emmanuel J. Favaloro

**Affiliations:** 1 Institute of Clinical Pathology and Medical Research (ICPMR), Westmead Hospital, Westmead, NSW 2145, Australia; Benjamin.Reardon@health.nsw.gov.au (B.R.); leonardo.pasalic@health.nsw.gov.au (L.P.); 2 NSW Health Pathology, Westmead, NSW 2145, Australia; 3 Westmead Clinical School, University of Sydney, Westmead, NSW 2145, Australia; 4 Sydney Centres for Thrombosis and Haemostasis, Westmead Hospital, Westmead, NSW 2145, Australia; 5 School of Biomedical Sciences, Charles Sturt University, Wagga Wagga, NSW 2650, Australia

**Keywords:** von Willebrand factor, endothelium, cardiac failure, ADAMTS13

## Abstract

von Willebrand factor (VWF) is an adhesive protein involved in primary hemostasis and facilitates platelet adhesion to sites of vascular injury, thereby promoting thrombus formation. VWF exists in plasma as multimers of increasing size, with the largest (high molecular weight; HMW) expressing the greatest functional activity. A deficiency of VWF is associated with a bleeding disorder called von Willebrand disease (VWD), whereas an excess of VWF, in particular the HMW forms, is associated with thrombosis. ADAMTS13 (a disintegrin and metalloproteinase with thrombospondin type 1 motif-13), also known as VWF-cleaving protease, functions to moderate the activity of VWF by cleaving multimers of VWF and limiting the expression of the largest multimers of VWF. A deficiency of ADAMTS13 is therefore associated with an excess of (HMW forms of) VWF, and thus thrombosis. Indeed, any disturbance of the VWF/ADAMTS13 ratio or ‘axis’ may be associated with pathophysiological processes, including prothrombotic tendency. However, both thrombosis or bleeding may be associated with such disturbances, depending on the presenting events. This review evaluates the relationship of VWF and ADAMTS13 with cardiac disease, including cardiac failure, and associated pathophysiology.

## 1. Introduction

Cardiac failure is becoming increasingly common with improvements in therapeutic targets in an aging population [1]. Cardiac failure represents a clinical syndrome with significant morbidity and mortality. The pathophysiology of cardiac failure is a complex mix of structural and functional alterations. However, the exact mechanisms underlying the disease remain poorly defined [2]. Endothelial dysfunction has been identified as one of the components of cardiac failure pathophysiology, whereby disturbances in coronary microcirculation are thought to contribute to cardiac failure and its progression [3,4]. Inflammatory or ischemic endothelial activation results in the release of von Willebrand factor (VWF) from Weibel–Palade bodies held in endothelial cells [5]. VWF is a large, complex protein that has a crucial role in platelet adhesion and aggregation and is involved in both primary and secondary haemostasis [6]. VWF exists in plasma as multimers of increasing size, with the largest (high molecular weight; HMW) expressing the greatest functional activity. A deficiency of VWF is associated with a congenital bleeding disorder called von Willebrand disease (VWD). In addition, the loss of VWF can occur in a variety of acquired conditions [6,7]. Of note, certain cardiac lesions, particularly aortic stenosis, can elongate VWF multimers in the shear field, resulting in proteolytic loss of the highest molecular weight forms, leading to subsequent loss of VWF activity and resultant bleeding [8,9]. VWF activity is controlled through cleavage by a disintegrin and metalloproteinase with thrombospondin type 1 motif-13 (ADAMTS13), also known as VWF-cleavage protease. Elevation of VWF and potential reduction in ADAMTS13 essentially represent biomarkers of endothelial dysfunction, as most recently typified in COVID-19 (Coronavirus Disease 2019) [10].

Plasma VWF levels can be assessed by means of VWF antigen (VWF: Ag) [11,12] and/or a variety of activity assays [6,11,12]. The most common VWF: Ag assays comprise latex-enhanced immunoassays and enzyme-linked immunosorbent assays [11,12]. Activity assays for VWF include measurements of binding to platelet glycoprotein Ib (GPIb), collagen and factor VIII [11]. However, in the main, studies reporting a single VWF parameter report VWF: Ag levels. VWF:Ag levels reflect both active and inactive VWF. There are over 20 different commercial options for the measurement of VWF: Ag, and publications do not always report the method used [10]. ADAMTS13 can also be measured as an antigen assay using enzyme-linked immunosorbent assays [13], or as an activity assay, with FRETS-based assays being common [13], or by using chemiluminescence [14] among other procedures [13]. However, in the main, studies reporting a single ADAMTS13 parameter report activity levels. In total, there are over 20 different commercial options for measurement of ADAMTS13, and publications do not always report the method used [10].

Decreased ADAMTS13 and increased VWF levels have been shown to be contributory drivers in myocardial infarction [15]. Circulating endothelial cells have been shown to predict vascular events in patients with established coronary artery disease and affect myocardial infarct size following a myocardial ischaemic event [16,17,18]. Alterations in both VWF and ADAMTS13 levels have also been implicated in patients with atrial fibrillation (AF) [19,20]. This can be identified as a disturbance of the VWF/ADAMTS13 axis or an increase in the relative VWF/ADAMTS13 ratio [21]. ADAMTS13 is most commonly known for its deficiency state as part of thrombotic thrombocytopenic purpura (TTP), a prothrombotic disorder [13]. Although rare, acquired TTP has been described following cardiac surgery [22,23]. In this review, we aim to describe the associations of VWF, ADAMTS13, and cardiac disease in some detail.

## 2. Endothelial Dysfunction and Cardiac Disease

Normal vascular endothelium plays a multifaceted regulatory role in blood vessel function, including blood flow, prevention, and the propagation of thrombus at sites of vascular injury and both anti- and pro-inflammatory functions when appropriately stimulated. When the vascular endothelium becomes dysfunctional, this may result in abnormal coronary microcirculatory flow, impairing myocardial perfusion and therefore function [24]. The process may also result in more overt arterial thrombus formation, as seen in cerebrovascular and coronary arterial events [3]. Endothelial dysfunction, represented by circulating endothelial cells, is predictive of major adverse cardiac events and cardiac remodeling in patients after ST elevation myocardial infarction [25]. Endothelial dysfunction has been reported as an independent predictor of morbidity and mortality in patients with cardiac failure [26,27]. Endothelial dysfunction has been associated with a higher rate of cardiovascular events in patients with cardiac failure and a greater risk of atrial fibrillation in patients with non-obstructive coronary artery disease [28,29]. This may be a result of loss of nitric-oxide dependent vasodilatory signals, proinflammatory states that resulting from cardiac failure, as well as its prothrombotic properties [30].

VWF is a large multimeric glycoprotein selectively expressed in endothelial cells and megakaryocytes, and present in the subendothelial matrix, platelets and plasma. VWF is stored in cigar-shaped vesicles called Weibel–Palade bodies in endothelial cells [31]. Endothelial injury results in stimulation of Weibel–Palade bodies to secrete their contents including VWF into circulation. Due to blood shear, VWF then unfolds, binding the GPIb receptor of platelets to the A1-domain of VWF [32].

The involvement of VWF in local vascular injury and homeostasis lends itself to being a key determinant of endothelial dysfunction, and thus cardiac failure pathogenesis [33]. In a cohort of non-ischaemic, dilated cardiac failure patients with average symptom duration of 19 months, VWF RNA expression by real-time PCR on endomyocardial biopsy was demonstrated to be upregulated, suggesting that over time patients continue to present with progressive endothelial dysfunction despite treatment optimisation [3]. Plasma VWF:Ag levels have also been found to be substantially increased in patients with acute or recent decompensated cardiac failure [34]. If these elevations in VWF are persistent, this may result in a higher risk of thrombosis [35]. Increased plasma VWF:Ag has also been demonstrated as an independent predictor of long-term outcome in these patients [36].

In contrast, animal models have demonstrated that VWF deficiency is protective against atherosclerosis and arterial thrombotic risk [37]. Cohort data from patients with VWD have been conflicting, demonstrating that arterial thrombotic events still occur in patients with VWD. However, these seem to occur with a lower incidence than the general population [38,39]. Additionally, in patients with VWD, the risk of hypertension, a well-known risk factor of cardiovascular events, is reduced [40].

## 3. ADAMTS13, VWF and Cardiac Dysfunction

The multimeric size of VWF, and therefore its platelet binding activity, is regulated by cleavage by ADAMTS13. As noted above, ADAMTS13 deficiency is most associated with the rare condition of TTP. TTP is a thrombotic microangiopathy caused by severely reduced ADAMTS13 leading to platelet-rich thrombi, thrombocytopenia, and haemolytic anaemia. Acquired TTP after significant endothelial injury is now well recognized but considered a distinct clinical syndrome in the surgical field, particularly in cardiothoracic surgery [22]. The etiology of post-operative TTP may be secondary to autoimmune-mediated antibodies against ADAMTS13 [22,41]. Post-operative TTP following cardiothoracic surgery is associated with high patient morbidity and mortality [41,42]. Additionally, Le Besnerais et al. [42] showed that injecting ADAMTS13 knockout mice with recombinant VWF, leading to a TTP-like state, resulted in reduced left ventricular function, fractional shortening, and reduced cardiac output by day 2 after injection. This was associated with a decreased endothelial response to acetylcholine, indicative of early severe endothelial dysfunction [43].

ADAMTS13 has also been proposed as another biomarker of endothelial damage and dysfunction [19]. Low plasma ADAMTS13 activity has been shown to predict cardiac and cerebrovascular events in patients with established coronary artery disease [15,16,18]. Plasma ADAMTS13 activity has also been associated with myocardial infarct size and cardiac function after a myocardial ischaemic event [17]. Decreased activity of ADAMTS13 with concomitant high VWF:Ag levels has also been demonstrated as an independent predictor of clinical events in patients with cardiac failure [33]. Both VWF levels and ADAMTS13 activity have been correlated in older patients with atrial fibrillation (AF) and an increasing stroke risk calculator scoring system, the CHA2DS2-VASc (congestive heart failure, hypertension, age ≥ 75 years, diabetes mellitus, stroke/transient ischemic attack/thromboembolism, vascular disease, age 65–74 years, female) [44]. The mechanisms behind cerebral thrombo-embolism in patients with AF are not completely defined. However, it is clear that AF is associated with a prothrombotic state and higher VWF:Ag levels compared to healthy controls [45]. Zhang et al. further demonstrated that elevated VWF:Ag levels were independently associated with an elevated CHA2DS2-VASc score for stroke in patients with and without AF [19]. They also found that, in patients aged 65–74 years, patients with AF had elevated VWF levels and decreased ADAMTS13 activity compared to those without AF. This difference was not seen in patients aged ≥ 75 years, suggesting that AF is one of many factors affecting VWF levels and ADAMTS13 activity, and that age is an important factor affecting endothelial function. Decreased ADAMTS13 activity has also been implicated in the recurrent risk of AF in those undergoing cardioversion [20].

Plasma VWF:Ag levels are increased in coronary artery disease, ischaemic stroke, and venous thromboembolism, whereas ADAMTS13 activity levels are reduced [46]. This relationship between VWF and ADAMTS13 can be described as the VWF-ADAMTS13 axis and is indicative of vascular endothelial function [21]. The VWF-ADAMTS13 axis has been shown to be dysregulated in chronic thromboembolic pulmonary arterial hypertension, whereby increases in VWF, particularly compared to the level or activity of ADAMTS13, are seen, including following invasive intervention [47].

## 4. Angiogenesis and Acquired von Willebrand Disease

Although VWF is best known for its role in hemostasis, it has also recently been shown to be a key regulator of angiogenesis [48]. VWF is present in endothelial cells, plasma, and subendothelium, whereby its release leads to the initial adhesion of platelets to collagen. VWF also binds to factor VIII in circulation in its inactive state. The binding activity of VWF is determined by both its multimeric size and conformation [49]. In vitro studies of endothelial cells have shown that the inhibition of VWF expression by siRNA resulted in angiogenesis [48]. In VWF deficient mice, angiogenesis and vascular density were increased in several in vivo models [48]. A loss of balance between blood vessel proliferation and stabilization by surrounding extracellular matrix can lead to dysfunctional vessel formation, such as that found in angiodysplastic lesions. Up to 20% of patients with VWD, the most common inherited bleeding disorder in humans, commonly present with gastrointestinal bleeding from such lesions [50]. This can be severe and non-responsive to VWF replacement therapies. VWF deficiency has also been shown to enhance VEGFR-2 mediated endothelial migration and proliferation [48].

VWD can be classified as inherited, which can be further categorized into six different types (1, 2A, 2B, 2M, 2N, 3), and which results from a variety of mutations occurring throughout the *VWF* gene, or acquired, from a variety of conditions, including malignant disorders, aortic valve stenosis, or left ventricular assist devices [6,11,51,52]. Acquired VWD is more commonly called acquired von Willebrand syndrome (AVWS). Although mentioned as a rare disorder in the literature, AVWS is likely more common than recognized, given that milder forms may not manifest until a significant haemostatic challenge [52]. Acquired VWD has multifactorial etiological mechanisms based on the underlying disorder driving it [52]. This is summarized in Table 1.

Cardiovascular disorders form the largest group of disorders among the underlying pathogenic conditions associated with acquired VWD, with the two major groups being aortic stenosis associated gastrointestinal dysplasia and patients with left ventricular assist device (LVAD) or extracorporeal life support [52,53,54,55]. Shear stress-induced reduction of HMW VWF multimers and proteolysis of VWF as it passes through the stenotic valve, increased cleavage by ADAMTS13 and shear-induced VWF binding to platelets are the proposed mechanisms for AVWS in this setting [56]. This has been demonstrated in in vivo models [56]. Aortic valve replacement, both surgical and transcatheter-based, is often a definitive treatment for gastrointestinal bleeding, particularly from angiodysplastic lesions, leading to recovery of HMW VWF multimers [57]. Rarely, the association with regurgitant valvular lesions is seen including reported regurgitation secondary to (infectious) endocarditis [58]. In the instance of LVAD or extracorporeal life support, it is the device-related increase in shear stress, resulting in similar conditions of aortic stenosis [55]. Other proposed mechanisms of ventricular assist device induced AVWS include proteolytic degradation of VWF and excess platelet activation leading to adsorption of HMW VWF [8,59]. Patients with ventricular assist devices and AVWS who undergo cardiac transplantation and LVAD removal are cured of their bleeding phenotype [9]. Re-engineering of devices may be able to mitigate this problem to some extent.

## 5. Potential Therapeutic Targets

VWF is an attractive therapeutic target in the treatment of acute vascular events as well as secondary prophylaxis in select groups. VWF, including its production and binding to GPIb, collagen and Glycoprotein IIbIIIa, as well as ADAMTS13, have been well recognized as potential therapeutic targets within management of vascular events and endothelial dysfunction. Targeting a reduction in the size of Weibel–Palade bodies, the production site of VWF, has been shown to inhibit VWF pro-haemostatic potential [60]. This may be a potential adjunctive therapy to anti-thrombotic therapies in arterial vascular events, or also for the anti-thrombotic therapy of disseminated intravascular coagulation, which has also been found to be associated with high levels of VWF [61]. In vitro analysis of VWF antagonist therapies demonstrated a time-dependent and dose-dependent effect on VWF activity and platelet aggregation studies, which was then shown to prevent arterial occlusive events in monkey models [62,63]. Recombinant ADAMTS13 administration has been shown to reduce infarct volume when used in combination with thrombolysis in mice models [64]. However, further research of such targets is needed within the field of cardiology in conjunction with current antithrombotic therapies, as well as in non-ischemic cardiac dysfunction.

## 6. Conclusions

**Figure 1:**
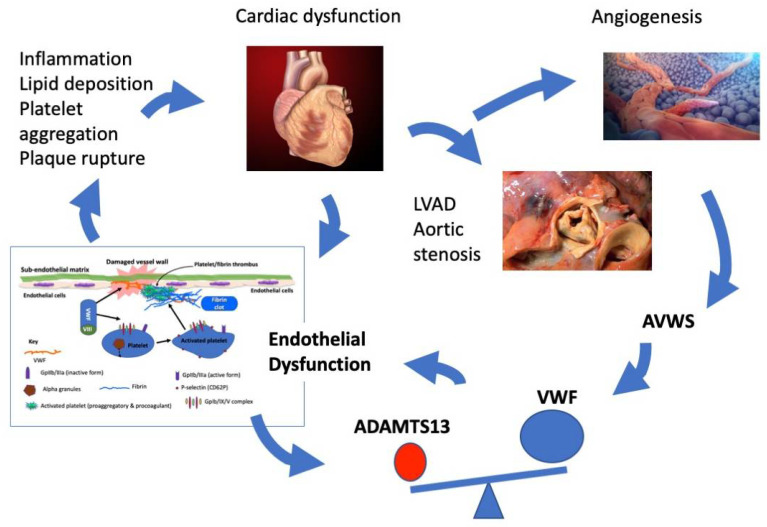
Legend. Original figure from the authors outlining some of the concepts discussed in the review. Abbreviations: ADAMTS13, a disintegrin and metalloproteinase with thrombospondin type 1 motif-13; AVWS, acquired von Willebrand syndrome; LVAD, left ventricular assist devices; VWF, von Willebrand factor. Portions of figures taken as follows, all with creative commons attributions: (a) Heart picture: Patrick J. Lynch; medical illustrator; C. Carl Jaffe; MD; cardiologist; Yale University Center for Advanced Instructional Media Medical Illustrations by Patrick Lynch, generated for multimedia teaching projects by the Yale University School of Medicine, Center for Advanced Instructional Media, 1987–2000. Patrick J. Lynch, http://patricklynch.net Creative Commons Attribution 2.5 License 2006; no usage restrictions except please preserve our creative credits: Patrick J. Lynch, medical illustrator; C. Carl Jaffe, MD, cardiologist. https://creativecommons.org/licenses/by/2.5/ (a) Aortic stenosis picture: Centers for Disease Control and Prevention’s Public Health Image Library (PHIL), with identification number #848. This image is a work of the Centers for Disease Control and Prevention, part of the United States Department of Health and Human Services, taken or made as part of an employee’s official duties. As a work of the U.S. federal government, the image is in the public domain. (b) Angiogenesis picture: https://www.scientificanimations.com/wiki-images/. Creative Commons Attribution-Share Alike 4.0 International (https://creativecommons.org/licenses/by-sa/4.0/deed.en) license.

As shown in this review, and as summarized in Figure 1, both VWF and ADAMTS13 can be implicated in the pathophysiology of cardiac disease and cardiac failure. This may lead to an increased risk of thrombosis where the VWF–ADAMTS13 axis is increased, reflecting a relative increase of VWF and/or a relative decrease in ADAMTS13 activity, with acquired TTP being the most extreme example. In contrast, disturbances whereby the VWF–ADAMTS13 axis reflects a relative decrease of VWF also reflect a risk factor for bleeding, with AVWS being the most extreme example [13]. We recommend that workers in the field of cardiac disease take a greater interest in both VWF and ADAMTS13 [21], to both identify the opposing risks of bleeding and thrombosis, and to potentially consider supportive therapies or curative approaches, as the case may require. Such strategies may include targeting the upregulated VWF or decreased ADAMTS13 activity in patients with cardiac failure, in order to halt persistent endothelial dysfunction and disease progression. In patients with AVWS, a different approach is needed, including supportive therapies with VWF [6,8].

## Figures and Tables

**Table 1:** Underlying disorders associated with acquired von Willebrand Disease.

**Lymphoproliferative Disorders**	Monoclonal Gammopathy of Undetermined Significance
	Multiple Myeloma
	Non-Hodgkin’s Lymphoma
	Waldenstrom’s Macroglobulinemia
	Hairy Cell Leukemia
**Myeloproliferative Disorders**	Essential Thrombocythemia
	Polycythemia Vera
	Chronic Myeloid Leukemia
**Tumours**	Wilms’ Tumor
	Ewing’s Sarcoma
**Cardiac Disorders**	Aortic Stenosis
	Left Ventricular Assist Devices
	Heart Transplantation
	Coronary Artery bypass Surgery
	Paravalvular Leak
	Hypertrophic Obstructive Cardiomyopathy
	Congenital Heart Disease
**Autoimmune**	SYSTEMATIC Lupus Erythematosus
	Other Autoimmune Disorders
**Drug Induced**	Cefotaxime
	Levofloxacin
	Ciprofloxacin
	Valproic Acid
	Hydroxy Ethyl Starch
	High-dose Recombinant Factor VIII
**Miscellaneous**	Gaucher’s Disease
	Renal Transplantation
	Hypothyroidism
	Extracorporeal Membrane Oxygenation Devices

Adapted from Colonne et al. [6] and Shetty et al. [52].

## Data Availability

The review does not report any new data.
